# *Batrachochytrium salamandrivorans* (Bsal) not detected in an intensive survey of wild North American amphibians

**DOI:** 10.1038/s41598-020-69486-x

**Published:** 2020-08-03

**Authors:** J. Hardin Waddle, Daniel A. Grear, Brittany A. Mosher, Evan H. Campbell Grant, Michael J. Adams, Adam R. Backlin, William J. Barichivich, Adrianne B. Brand, Gary M. Bucciarelli, Daniel L. Calhoun, Tara Chestnut, Jon M. Davenport, Andrew E. Dietrich, Robert N. Fisher, Brad M. Glorioso, Brian J. Halstead, Marc P. Hayes, R. Ken Honeycutt, Blake R. Hossack, Patrick M. Kleeman, Julio A. Lemos-Espinal, Jeffrey M. Lorch, Brome McCreary, Erin Muths, Christopher A. Pearl, Katherine L. D. Richgels, Charles W. Robinson, Mark F. Roth, Jennifer C. Rowe, Walt Sadinski, Brent H. Sigafus, Iga Stasiak, Samuel Sweet, Susan C. Walls, Gregory J. Watkins-Colwell, C. LeAnn White, Lori A. Williams, Megan E. Winzeler

**Affiliations:** 1 Wetland and Aquatic Research Center, U.S. Geological Survey, Gainesville, FL 32653 USA; 2 National Wildlife Health Center, U.S. Geological Survey, Madison, WI 53711 USA; 3 Department of Ecosystem Science and Management, Pennsylvania State University, University Park, PA 16802 USA; 4 Patuxent Wildlife Research Center, U.S. Geological Survey, Turners Falls, MA 01376 USA; 5 Rubenstein School of Environment and Natural Resources, University of Vermont, Burlington, VT 05405 USA; 6 Forest and Rangeland Ecosystem Science Center, U.S. Geological Survey, Corvallis, OR 97330 USA; 7 Western Ecological Research Center, U.S. Geological Survey, San Diego, CA 92101 USA; 8 Department of Ecology and Evolutionary Biology, UCLA La Kretz Center for California Conservation Science, University of California Los Angeles, Los Angeles, CA 90095 USA; 9 South Atlantic Water Science Center, U.S. Geological Survey, Norcross, GA 30093 USA; 10 Mount Rainier National Park, Ashford, WA 98304 USA; 11 Department of Biology, Appalachian State University, Boone, NC 28608 USA; 12 Wetland and Aquatic Research Center, U.S. Geological Survey, Lafayette, LA 70506 USA; 13 Western Ecological Research Center, U.S. Geological Survey, Dixon, CA 95620 USA; 14 Washington Department of Fish and Wildlife, Olympia, 98501 USA; 15 Northern Rocky Mountain Science Center, U.S. Geological Survey, Missoula, MT 59801 USA; 16 Western Ecological Research Center, U.S. Geological Survey, Point Reyes, CA 94956 USA; 17 Laboratorio de Ecología UBIPRO, FES Iztacala UNAM, 54090 Tlalnepantla, State of Maxico Mexico; 18 Fort Collins Science Center, U.S. Geological Survey, Fort Collins, CO 80526 USA; 19 Upper Midwest Environmental Sciences Center, U.S. Geological Survey, La Crosse, WI 54603 USA; 20 Southwest Biological Science Center, U.S. Geological Survey, Tucson, AZ 85721 USA; 21 Saskatchewan Ministry of Environment, 112 Research Drive, Saskatoon, SK S7N 3R3 Canada; 22 Department of Ecology, Evolution, and Marine Biology, University of California Santa Barbara, Santa Barbara, CA 93106 USA; 23 Peabody Museum of Natural History, Yale University, New Haven, CT 06520 USA; 24 North Carolina Wildlife Resources Commission, Fletcher, NC 28732 USA; 25 Savannah River Ecology Laboratory, University of Georgia, Aiken, SC 29802 USA

**Keywords:** Ecological epidemiology, Herpetology

## Abstract

The salamander chytrid fungus (*Batrachochytrium salamandrivorans* [Bsal]) is causing massive mortality of salamanders in Europe. The potential for spread via international trade into North America and the high diversity of salamanders has catalyzed concern about Bsal in the U.S. Surveillance programs for invading pathogens must initially meet challenges that include low rates of occurrence on the landscape, low prevalence at a site, and imperfect detection of the diagnostic tests. We implemented a large-scale survey to determine if Bsal was present in North America designed to target taxa and localities where Bsal was determined highest risk to be present based on species susceptibility and geography. Our analysis included a Bayesian model to estimate the probability of occurrence of Bsal given our prior knowledge of the occurrence and prevalence of the pathogen. We failed to detect Bsal in any of 11,189 samples from 594 sites in 223 counties within 35 U.S. states and one site in Mexico. Our modeling indicates that Bsal is highly unlikely to occur within wild amphibians in the U.S. and suggests that the best proactive response is to continue mitigation efforts against the introduction and establishment of the disease and to develop plans to reduce impacts should Bsal establish.

## Introduction

The salamander chytrid fungus (*Batrachochytrium salamandrivorans* [Bsal]) is causing massive mortality of fire salamanders in middle Europe^1^. There is considerable concern that Bsal is likely to be introduced into North America with severe consequences. Concern is based on the susceptibility of several families of salamanders in the United States (U.S.) to Bsal infections^2^, the designation of the eastern and northwestern regions of the U.S. as salamander hotspots^3–4^, and the ease of transport to the U.S. via the pet trade or other international trade^5^. Bsal may already be present in the United States, but undetected because of the sheer volume of imported salamander species known to be carriers of the pathogen^6–7^. Bsal was not detected in a sample of 639 pet salamanders in the U.S.^8^, however, determining presence or occurrence of Bsal in captive populations is difficult because the number and distribution of captive hosts is unknown. If Bsal becomes established in the U.S., the effects on global salamander biodiversity could be devastating. Modeling suggests that there are portions of the U.S. with high habitat suitability and invasion potential for Bsal because of climate, species richness of salamanders, and proximity to major ports of entry^6–7^. Accordingly, the U.S. Fish and Wildlife Service invoked the Lacey Act to declare 201 species of salamander injurious (known to be a member of a genus containing at least one species susceptible to Bsal), resulting in the barring of international transport^9^. A similar ban on the trade in salamanders was also enacted in Canada^10^. Although interstate transport of these salamanders was originally banned under the Lacey Act, a 2017 decision by the U.S. Court of Appeals for the District of Columbia held that there was no ban on interstate transport within the continental U.S.

The early detection of invading pathogens presents several difficulties to surveillance programs. First, detection is difficult, despite large sample sizes^11^, when pathogens are present but rare on the landscape, or when site-level prevalence is low within host populations. Second, the spatial extent of sampling needs to be large for pathogens without discrete points of likely introduction. Finally, imperfect diagnostic testing and a large surveillance effort can lead to false positives or imprecision in inference from few positive samples^12^ which need to be interpreted carefully.

Because of significant concern about the effect of Bsal on the salamander fauna of the U.S.^13–14^, we implemented a survey for this pathogen across the contiguous U.S. We designed a sampling scheme that considered geographic risk^7^ and species susceptibility^2^. Our objective was to maximize the probability of detecting Bsal if it was present in wild salamander populations. Specifically, we evaluated the probability that Bsal was present at sampled sites under a suite of invasion scenarios. This effort was designed to reduce uncertainty about where Bsal might be present and provide information to facilitate responses to the threat of invasion by this lethal pathogen. We also discuss optimal sampling designs for low occurrence and low prevalence pathogens and how our survey results contribute to next generation strategies to identify and mitigate emerging infectious diseases.

## Results

**Figure 1:**
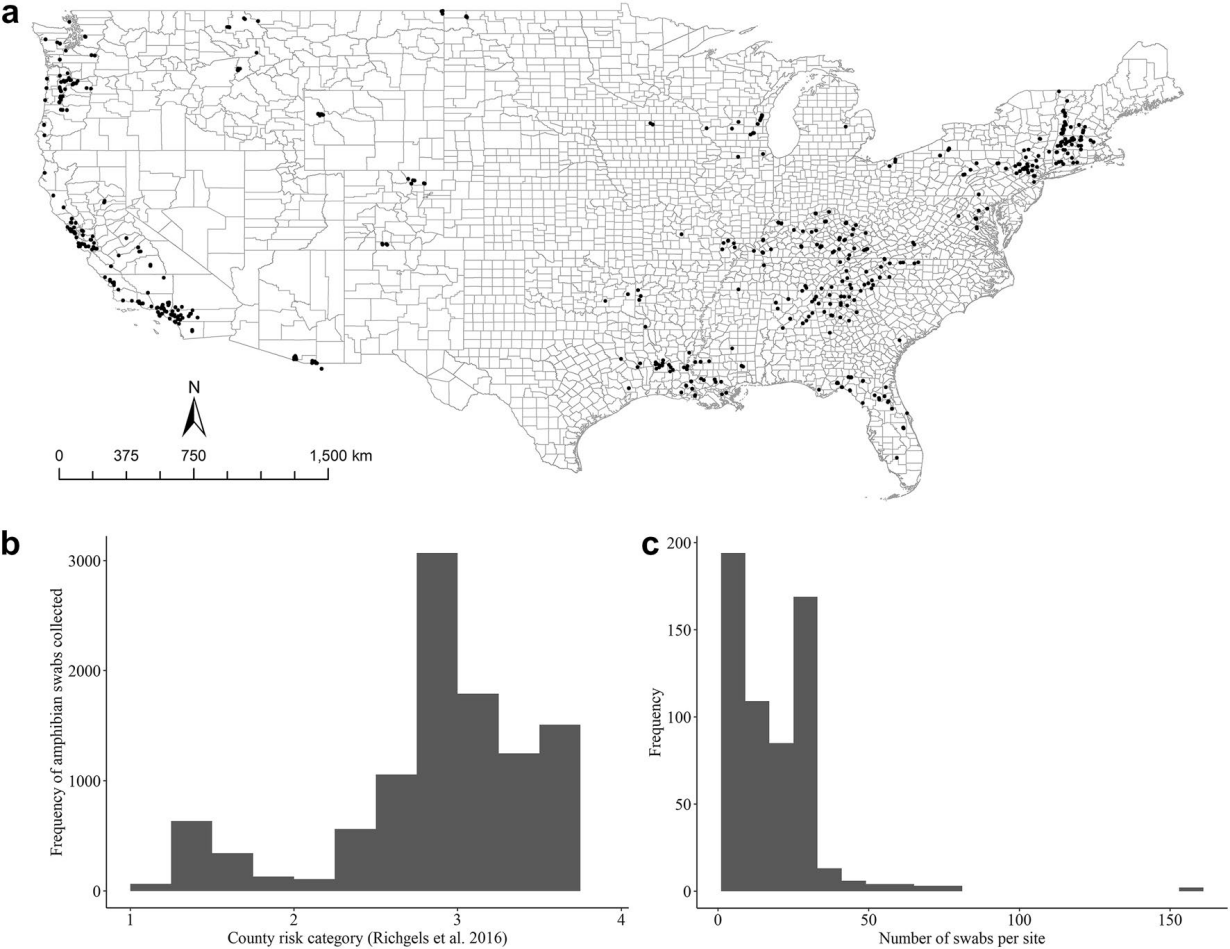
(**a**) Map of the contiguous U.S. with all 594 sites sampled for Bsal represented as circles. (**b**) Histogram of the frequency of sampled sites across the risk of Bsal at the county level assigned by Richgels et al.^7^. (**c**) Frequency histogram of number of Bsal samples collected per site.

We collected 11,189 samples from 54 species across 594 sites in 223 counties within 35 U.S. states and one site in Mexico (Fig. 1a). Samples were collected between May 2014 and August 2017. Bsal was not detected in any sample (Supplement). Our sampling was directed at higher risk category areas with 52% of sampled sites in areas ranked in the highest one-third of Richgels et al.^7^ risk scores, 38% in the middle one-third, and 10% in the lowest one-third (Fig. 1b). The average number of individuals sampled per site was 18.6 (range: 1 to 165 individuals), and we sampled at least 30 individuals (i.e., had > 90% cumulative detection probability; Eq. (1) at 184/594 sites, given a 10% prevalence (Fig. 1c).

**Figure 2:**
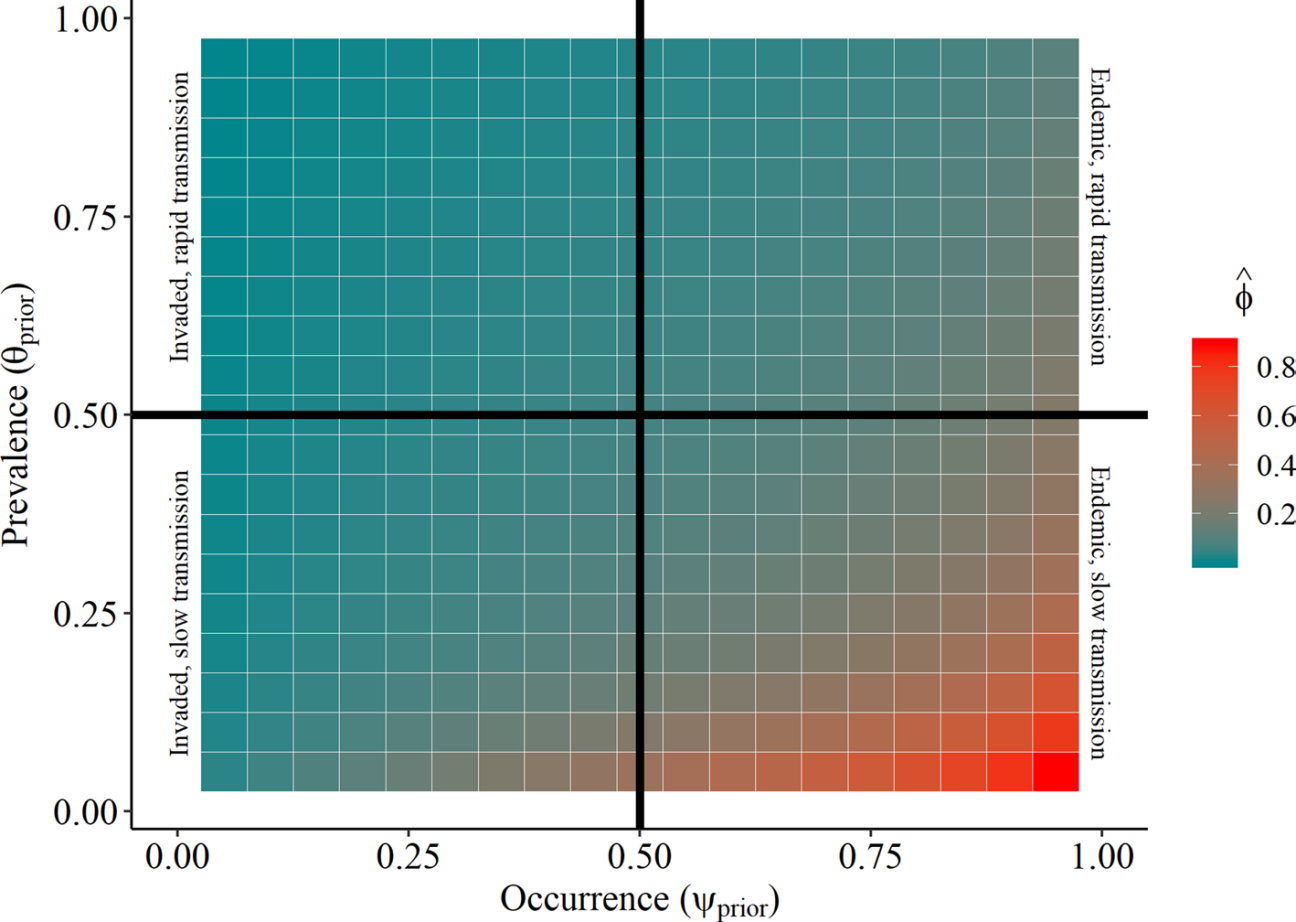
Estimated probability of Bsal occurrence conditional on non-detection ($\hat{\varphi }$) for the range of prior expectations about Bsal occupancy ($\psi_{prior}$) and prevalence ($\theta_{prior}$).

Given our sampling effort and a Bsal detection probability assumption of 0.75, our non-detection data indicated that Bsal was very unlikely to occur at any of our sampled sites ($\hat{\varphi } \approx 0$) under most of our invasion hypotheses (Fig. 2). However, our sampling effort was insufficient to confirm Bsal absence at any other randomly selected sites for part of the high occupancy, low prevalence sample space (Fig. 2).

## Discussion

Emerging infectious diseases pose a major threat to wildlife populations^15^, and amphibians are particularly vulnerable^16^. Surveillance designed around risk, ecological hypotheses of invasion, and specific objectives provide information about the probability of occurrence of a pathogen. We used a spatial quantitative risk model^7^ to design a surveillance sample that allowed us to refine our expectation of the occurrence of Bsal in the U.S. We sampled proportional to county level risk and failed to detect the pathogen in any of 11,189 samples. We conclude that, unless Bsal is both extremely widespread and at low prevalence within populations, it is highly unlikely to occur at any of our sampled sites. We also predict that if prevalence of Bsal is high, as would be expected from locations in Europe^17^ or Asia^18^, then it is unlikely that Bsal occurs within our sampled areas (U.S. counties) unless its occurrence is so rare that the probability of sampling a Bsal-occupied site is extremely low. We note that another study also found no evidence of Bsal in North America^19^. We also note that state of Bsal occurrence following the European introduction suggests that a low prevalence, high occurrence state is unlikely^1–2^. Nonetheless, we do not think making strong inference about the likelihood of potential post-introduction states in North America, based on Europe, is appropriate because of the large difference in potential host richness and scale in North America.

Our data are consistent with the evidence that suggests North America is naïve to Bsal. We cannot rule out recent introduction to a site we did not sample nor introduction at the time of sampling, but it is reasonable to assume Bsal is absent or very rare in North America. Our confidence in this conclusion is dependent on two assumptions—first, that our risk model^7^ correctly ranks counties in their invasion potential, and second, an assumption of high prevalence of Bsal at sites where it occurs. Research on host susceptibility to Bsal in amphibian species indigenous to North America^20^ will provide important information to estimate more precisely what occurrence and prevalence scenario is most likely to manifest if Bsal is introduced. A better understanding of likely invasion routes and the prevalence of Bsal at these entry sites will assist in designing efficient surveillance by determining how best to distribute resources towards sampling more intensely (more samples per site) or extensively (greater spatial coverage).

These results provide a single observation of the state of occurrence of Bsal in the U.S. However, the system is dynamic (i.e., the risk of introduction to the wild of an infected individual or material exists—Bsal has not been eliminated from potential introduction, and an ongoing surveillance program will continue to provide information to managers concerned with preventing the introduction, establishment and spread of Bsal). We suggest continued surveillance with a focus on the following potential invasion pathways (1) occurrence and prevalence of Bsal infection in amphibian species known to be susceptible to Bsal but are outside the scope of the Lacey Act rule, (2) occurrence of Bsal within captive amphibians in North America, and (3) explicitly estimating occurrence (or conversely absence) of Bsal in wild amphibians in localized areas of North America where the effects of Bsal, if introduced, would be most catastrophic. Additionally, the deployment of an accurate means of sampling the environment for Bsal rather than relying on detection of infected amphibians would be more efficient and provide a method to determine Bsal presence in the absence of amphibian hosts in cases where high lethality and quick spread leave no amphibians to sample^21–23^.

Our results suggest that there is still time to develop and implement proactive management to reduce the likelihood of introduction and establishment and impacts of invasion should it occur, and to plan a response for when Bsal is first detected in North America^13–14^. Remaining tasks include the implementation of ongoing, focused surveillance monitoring, and development and testing of management strategies and interventions to mitigate the likelihood of introduction and establishment of Bsal and severity of expected negative outcomes if Bsal is established in the U.S. (i.e., state-dependent decisions which may be informed by a surveillance program).

## Methods

### Field sampling

We defined a sampling universe based on the presence of non-zero estimated introduction risk in the contiguous United States^7^ within the range of salamander species known to be susceptible to Bsal^2^. Because of their presumed high susceptibility to Bsal^2^ and large ranges^24^, we targeted newts of the genera *Notophthalmus* and *Taricha* in the eastern and western U.S., respectively. Challenge trials for most of the diverse amphibian fauna in the U.S. were lacking when we designed the study, though we expected that some anuran species can serve as infection reservoirs of Bsal^25–26^. We therefore sampled other amphibian species (anurans and caudates) as well (Supplement).

We set a target of 10,000 samples across the United States. Our expectation was that if Bsal was introduced into the U.S. it would most likely be transmitted by an infected individual intentionally released. Site selection was therefore non-random as we sought sites that were generally accessible to the public or near areas frequented by visitors. Accordingly, we avoided remote areas that would be less prone to such an introduction. We defined a site as a waterbody, wetland, or group of proximate aquatic habitats that could reasonably be epidemiologically linked based on the transmission of Bsal by annual host movements or transport of infective stages via water. We aimed to capture 30 animals per site (*N*), which would result in 90% certainty of detecting Bsal when present, assuming a Bsal detection probability (*p*) of 0.75 on an infected individual^27^ and a presumed low prevalence value of 0.10. We recognize that a prevalence value lower than 0.10 may be possible, but it would have been prohibitively difficult to sample more individuals per site.

1 $$ Certainty = 1 - [\theta *(1 - p) + (1 - \theta )]^{N} $$

We captured target animals by hand, net, or trap. After capture, we handled each animal separately using disposable, powderless vinyl gloves and new, clean plastic bags to avoid cross contamination. All handling of animals was conducted in accordance with relevant guidelines and with appropriate collecting permits. All experimental protocols were approved by U.S. Geological Survey Institutional Animal Care and Use Committee. Appropriate permit numbers and information may be obtained from first author upon request. We rubbed rayon-tipped sterile swabs (MW-113, Medical Wire & Equipment, Corsham, England) over the plantar side of one front and one hind limb, the ventral tail surface of caudates, the dorsal side of the body, and the ventral surface of the body 5 times each^28^. We placed the swabs into sterile plastic vials with 20 μl of sterile deionized water. We recorded the snout-vent length, sex, and any visible signs of skin lesions for each individual. We collected two separate swabs from each animal, holding one in reserve to provide confirmation if Bsal was detected on the first swab. We chilled swabs immediately after field collection and subsequently froze them at ≤ − 20 °C within 3 days. Frozen swabs were sent to the U.S. Geological Survey’s National Wildlife Health Center in Madison, Wisconsin, for analysis.

### Molecular methods

We extracted DNA from swabs as described by Hyatt et al.^29^ except that 125 μl of PrepMan® Ultra Sample Preparation Reagent (Applied Biosystems, Foster City, CA) and 100 mg of zirconium/silica beads (Biospec Products, Bartlesville, OK) were used so that the entire swab was immersed. The bead-beating steps were conducted using a FastPrep®-24 homogenizer (MP Biomedicals, Santa Ana, CA). We used a real-time TaqMan polymerase chain reaction (PCR) for detection of Bsal on the extracted DNA as described in Blooi et al.^30–31^. We ran reactions on the 7,500 fast real-time PCR system (Applied Biosystems, Foster City, CA) using QuantiFast Probe RT-PCR mastermix kit with ROX dye (Qiagen, Valencia, CA) and BSA as per the kit instructions. We used five microliters of the PrepMan® solution containing the extracted DNA as template for the PCR. We included a negative extraction control and a standard curve run in duplicate on each PCR plate. The standard curve consisted of five different concentrations of the target sequence for Bsal inserted into plasmids. The concentrations of the standards occurred at ten-fold dilutions ranging from 110–1,100,000 copies (0.5–5,000 fg DNA) per reaction (on some initial runs, the standard range was 11–110,000 copies per reaction). The threshold for signal detection was set at 5% of the maximum fluorescence of the standards run for that assay. We considered a positive detection of Bsal DNA if a detectable signal existed at 37 or fewer PCR cycles and no detection in all other cases. We calculated the efficiency of each run using standard curve amplification and repeated PCR plates with an efficiency of less than 90% or greater than 110%.

### Data analyses

The probability of failing to detect a species given that it occurs is different than the probability of occurrence given non-detection^32^. We focused on this latter quantity and estimated the average probability of Bsal occurrence at sampled sites, given non-detection data, survey effort, and alternative hypotheses about the status of Bsal in the U.S. We defined occupancy as the probability of Bsal occurrence at the site level and prevalence as the probability of Bsal occurrence on an individual. Under this latter definition, prevalence included both infections and Bsal zoospores from the environment that might be detected on the skin of an infected individual. The probability of Bsal occurrence given non-detection was represented probabilistically as Pr(*z*_*i*_ = 1|Σ(*y*_*ij*_) = 0), where *z*_*i*_ is the latent occupancy state for site *i* (*z*_*i*_ = 1 for occupied sites and *z*_*i*_ = 0 for unoccupied sites) and *y*_*ij*_ is the imperfectly observed pathogen status of a sampled individual *j* at site *i*. At occupied sites, observations were a product of the pathogen status of the individual (*w*_*j*_ = 1 for pathogen positive individuals and *w*_*j*_ = 0 for pathogen negative individuals) and the probability of detecting Bsal on infected individuals (*p*).

Using Bayes Theorem, the probability of Bsal occurrence at a single site *i* conditional on non-detection ($\varphi_{i}$) can be calculated using prior expectations about Bsal occupancy ($\psi_{prior}$) and prevalence ($\theta_{prior}$). In addition, the total number of individuals sampled at each site (*N*) and the total number of replicates collected per individual (K) were considered.

2 $$ \begin{aligned} \varphi_{i} = \Pr {(}z_{i} = 1{|}\sum y_{ij} = 0) & = \frac{{\Pr {(}\sum y_{ij} = 0{|}z_{i} = 1){\Pr}\left( {z_{i} = 1} \right)}}{{\Pr {(}\sum y_{ij} = 0{|}z_{i} = 1){\Pr}\left( {z_{i} = 1} \right) + {\Pr}\left( {z_{i} = 0} \right)}} \\ & = \frac{{\left( {\left( {1 - \theta } \right) + \theta \left( {1 - p} \right)^{K} } \right)^{N} \psi }}{{\left( {\left( {1 - \theta } \right) + \theta \left( {1 - p} \right)^{K} } \right)^{N} \psi + \left( {1 - \psi } \right)}} \\ \end{aligned} $$

This equation yields the probability that an observation of Bsal non-detection came from an occupied site (i.e., a false negative), given survey effort (K, N) and prior expectations about pathogen detectability ($p$), occurrence ($\psi$) and prevalence ($\theta$).

Prior expectations were derived from four hypotheses about Bsal invasion in the United States using this probabilistic framework (Table 1), leading to different predictions about Bsal occurrence and prevalence. If Bsal is endemic to the U.S., we expect it to be widespread within suitable habitats (high $\psi$). If Bsal invaded the U.S. recently, we expect it to be present at a small proportion of locations (low $\psi$). This hypothesis is unlikely biologically given what we know about Bsal and invasive pathogens and it is only included here for theoretical completeness. We would expect that additional extensive sampling would fail to increase the posterior probability of this state of nature. In addition, and independent of occurrence rates, Bsal transmission within an infected population may vary. Reported Bsal prevalence values from field studies range across species, sites, and with time since invasion^17–18^. Therefore, we also consider two categories of site prevalence: a rapid transmission scenario where Bsal prevalence is high within infected populations (high $\theta$), and a slow transmission scenario ($\theta$) where Bsal prevalence is low within infected populations. To evaluate the probability that Bsal was present at any of our sampled sites given non-detection, we calculated Eq. (2) for each site across a range of occurrence ($\psi \,$= 0.05–0.95) and prevalence ($\theta \,$= 0.05–0.95) values and used the mean result of Eq. (2) ($\hat{\varphi }$) from all our sampled sites as the metric to summarize the probability of Bsal presence in our sampling frame.

**Table 1:** Hypotheses concerning the arrival and occurrence of *Batrachochytrium salamandrivorans* within sites ($\psi$) and populations ($\theta$), given it occurs in the United States.

	Occupancy ($\psi$)	
	Low	High
**Prevalence **$\left( {\varvec{\theta}} \right)$		
High	Recently invaded, rapid transmission	Endemic, rapid transmission
Low	Recently invaded, slow transmission	Endemic, slow transmission

## Supplementary information

Supplementary Information.

## Data Availability

All survey data used in this study have been deposited in ScienceBase and are accessible to the public^33^.
