# Supplementary material for: Batrachochytrium salamandrivorans (Bsal) not detected in an intensive survey of wild North American amphibians
Source: Sci Rep. 2020 Aug 3;10:13012. doi: 10.1038/s41598-020-69486-x (PMC7400573; doi:10.1038/s41598-020-69486-x)
Supplement: Supplementary file 1 — Supplementary Information. [file 41598_2020_69486_MOESM1_ESM.docx]

**Title:** *Batrachochytrium salamandrivorans* (Bsal) not detected in an intensive survey of wild North American amphibians

**Authors:**

J. Hardin Waddle^1^*

Daniel A. Grear^2^

Brittany A. Mosher^3,4,5^

Evan H. Campbell Grant^4^

Michael J. Adams^6^

Adam R. Backlin^7^

William J. Barichivich^1^

Adrianne B. Brand^4^

Gary M. Bucciarelli^8^

Daniel L. Calhoun^9^

Tara Chestnut^10^

Jon M. Davenport^11^

Andrew E. Dietrich^4^

Robert N. Fisher^7^

Brad M. Glorioso^12^

Brian J. Halstead^13^

Marc P. Hayes^14^

R. Ken Honeycutt^15^

Blake R. Hossack^15^

Patrick M. Kleeman^16^

Julio A. Lemos-Espinal^17^

Jeffrey M. Lorch^2^

Brome McCreary^6^

Erin Muths^18^

Christopher A. Pearl^6^

Katherine L. D. Richgels^2^

Charles W. Robinson^2^

Mark F. Roth^19^

Jennifer C. Rowe^6^

Walt Sadinski^19^

Brent H. Sigafus^20^

Iga Stasiak^21^

Samuel Sweet^22^

Susan C. Walls^1^

Gregory J. Watkins-Colwell^23^

C. LeAnn White^2^

Lori A. Williams^24^

Megan E. Winzeler^2,25^

*Corresponding author: waddleh@usgs.gov

^1^U.S. Geological Survey, Wetland and Aquatic Research Center, Gainesville, FL 32653, USA.

^2^U.S. Geological Survey, National Wildlife Health Center, Madison, WI 53711, USA.

^3^Pennsylvania State University, Department of Ecosystem Science and Management, University Park, PA 16802, USA.

^4^U.S. Geological Survey, Patuxent Wildlife Research Center, Turners Falls, MA 01376, USA.

^5^University of Vermont, Rubenstein School of Environment and Natural Resources, Burlington, VT 05405, USA.

^6^U.S. Geological Survey, Forest and Rangeland Ecosystem Science Center, Corvallis, OR 97330, USA.

^7^U.S. Geological Survey, Western Ecological Research Center, San Diego, CA 92101, USA.

^8^University of California Los Angeles, Department of Ecology and Evolutionary Biology, UCLA La Kretz Center for California Conservation Science, Los Angeles, CA 90095, USA.

^9^U.S. Geological Survey, South Atlantic Water Science Center, Norcross, GA 30093, USA.

^10^Mount Rainier National Park, Ashford, WA 98304, USA.

^11^Appalachian State University, Department of Biology, Boone, NC 28608, USA.

^12^U.S. Geological Survey, Wetland and Aquatic Research Center, Lafayette, LA 70506, USA.

^13^U.S. Geological Survey, Western Ecological Research Center, Dixon, CA 95620, USA.

^14^Washington Department of Fish and Wildlife, Olympia, WA 98501, USA.

^15^U.S. Geological Survey, Northern Rocky Mountain Science Center, Missoula, MT 59801, USA.

^16^U.S. Geological Survey, Western Ecological Research Center, Point Reyes, CA 94956, USA.

^17^FES Iztacala UNAM, Laboratorio de Ecología UBIPRO, Tlalnepantla, Estado de México 54090, México.

^18^U.S. Geological Survey, Fort Collins Science Center, Fort Collins, CO 80526, USA.

^19^U.S. Geological Survey, Upper Midwest Environmental Sciences Center, La Crosse, WI 54603, USA.

^20^U.S. Geological Survey, Southwest Biological Science Center, Tucson, AZ 85721, USA.

^21^Saskatchewan Ministry of Environment, 112 Research Drive, Saskatoon, SK S7N 3R3, Canada.

^22^University of California Santa Barbara, Department of Ecology, Evolution, and Marine Biology, Santa Barbara, CA 93106, USA.

^23^Yale University, Peabody Museum of Natural History, New Haven, CT 06520, USA.

^24^North Carolina Wildlife Resources Commission, Fletcher, North Carolina 28732, USA.

^25^University of Georgia, Savannah River Ecology Laboratory, Aiken, SC, 29802, USA.

**Supplement**

Table 1: Number of individuals of each amphibian species sampled and the number of individuals found to be positive for Bsal.

| **Species** | **No. Individuals Sampled** | **No. Positive for Bsal** |
| --- | --- | --- |
| **Caudates**  *Ambystoma annulatum* | 4 | 0 |
| *Ambystoma californiense* | 58 | 0 |
| *Ambystoma cingulatum* | 40 | 0 |
| *Ambystoma gracile* | 74 | 0 |
| *Ambystoma laterale* | 3 | 0 |
| *Ambystoma macrodactylum* | 173 | 0 |
| *Ambystoma maculatum* | 166 | 0 |
| *Ambystoma mavortium* | 411 | 0 |
| *Ambystoma opacum* | 26 | 0 |
| *Ambystoma talpoideum* | 125 | 0 |
| *Ambystoma tigrinum* | 85 | 0 |
| *Amphiuma means* | 2 | 0 |
| *Amphiuma tridactylum* | 10 | 0 |
| *Aneides lugubris* | 1 | 0 |
| *Aneides vagrans* | 1 | 0 |
| *Batrachoseps attenuatus* | 10 | 0 |
| *Batrachoseps gavilanensis* | 5 | 0 |
| *Batrachoseps major* | 53 | 0 |
| *Batrachoseps minor* | 12 | 0 |
| *Batrachoseps nigriventris* | 119 | 0 |
| *Batrachoseps pacificus* | 5 | 0 |
| *Batrachoseps robustus* | 1 | 0 |
| *Batrachoseps stebbinsi* | 4 | 0 |
| *Dicamptodon ensatus* | 102 | 0 |
| *Dicamptodon tenebrosus* | 30 | 0 |
| *Ensatina eschscholtzii* | 12 | 0 |
| *Eurycea bislineata* | 6 | 0 |
| *Eurycea cirrigera* | 22 | 0 |
| *Gyrinophilus porphyriticus* | 5 | 0 |
| *Necturus beyeri* | 14 | 0 |
| *Necturus maculosus* | 49 | 0 |
| *Notophthalmus perstriatus* | 173 | 0 |
| *Notophthalmus viridescens* | 5952 | 0 |
| *Plethodon cinereus* | 13 | 0 |
| *Plethodon glutinosus* | 13 | 0 |
| *Pseudotriton ruber* | 1 | 0 |
| *Siren intermedia* | 74 | 0 |
| *Taricha granulosa* | 1846 | 0 |
| *Taricha sierrae* | 136 | 0 |
| *Taricha sp.* | 42 | 0 |
| *Taricha torosa* | 592 | 0 |
| **Caudate Subtotal** | **10470** | **0** |
| **Anurans** |  |  |
| *Anaxyrus boreas* | 210 | 0 |
| *Anaxyrus punctatus* | 10 | 0 |
| *Lithobates catesbeianus* | 171 | 0 |
| *Lithobates chiricahuensis* | 164 | 0 |
| *Lithobates pipiens* | 1 | 0 |
| *Pseudacris cadaverina* | 9 | 0 |
| *Pseudacris maculata* | 21 | 0 |
| *Pseudacris regilla* | 2 | 0 |
| *Pseudacris triseriata* | 1 | 0 |
| *Rana aurora* | 1 | 0 |
| *Rana luteiventris* | 68 | 0 |
| *Rana muscosa* | 60 | 0 |
| *Spea hammondii* | 1 | 0 |
| **Anuran Subtotal** | **719** | **0** |
|  |  |  |
| **Amphibian Grand Total** | **11189** | **0** |
